# Supplementary material for: Evaluation of Non-Invasive Gargle Lavage Sampling for the Detection of SARS-CoV-2 Using rRT-PCR or Antigen Assay
Source: Viruses. 2022 Dec 19;14(12):2829. doi: 10.3390/v14122829 (PMC9786102; doi:10.3390/v14122829)
Supplement: Supplementary file 1 [file viruses-14-02829-s001.zip › Supplementary Files/Figure S2.pdf]

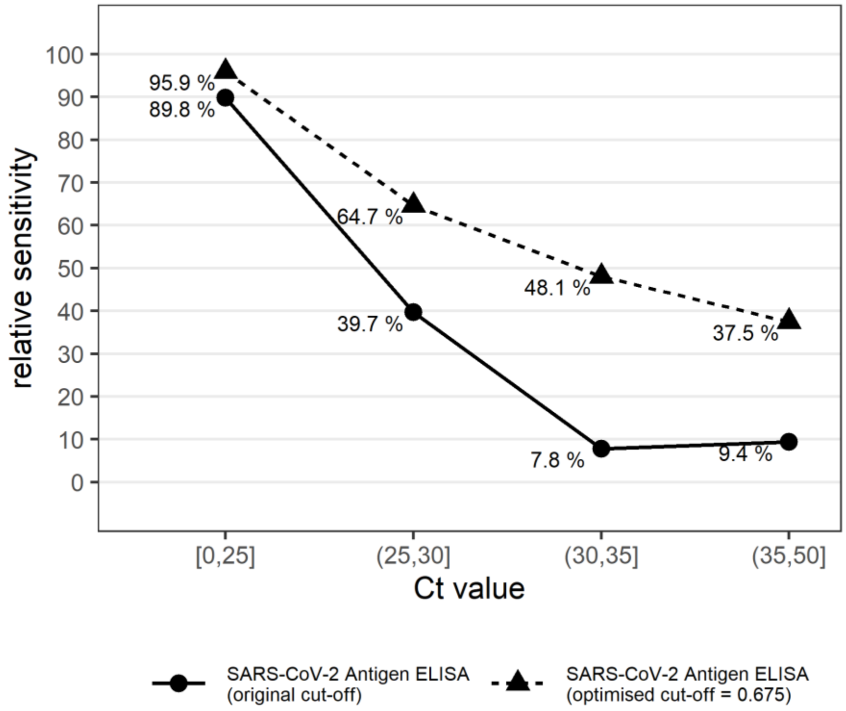

Figure S2. SARS-CoV-2 Antigen ELISA assay sensitivity for SARS-CoV-2 detection according to SARS-CoV-2 viral load in gargle lavage samples (*N* gene Ct value) using the original manufacturer's and optimised cut-off values.
