# Supplementary material for: Evaluation of Non-Invasive Gargle Lavage Sampling for the Detection of SARS-CoV-2 Using rRT-PCR or Antigen Assay
Source: Viruses. 2022 Dec 19;14(12):2829. doi: 10.3390/v14122829 (PMC9786102; doi:10.3390/v14122829)
Supplement: Supplementary file 1 [file viruses-14-02829-s001.zip › Supplementary Files/Table S1.pdf]

Table S1. Stability of SARS-CoV-2 in simulated gargle lavage samples and in GARGTEST medium dissolved in water.

			Day 0				Day 1				Day 4				Day 7				Day 11				Day 14			
			RdRP gene	*E* gene	*N* gene	*GAPDH* gene	*RdRP* gene	*E* gene	*N* gene	*GAPDH* gene	*RdRP* gene	*E* gene	*N* gene	*GAPDH* gene	*RdRP* gene	*E* gene	*N* gene	*GAPDH* gene	*RdRP* gene	*E* gene	*N* gene	*GAPDH* gene	*RdRP* gene	*E* gene	*N* gene	*GAPDH* gene
	Temperature (°C)	Viral load (PFU/ml)	ct	ct	ct	ct	ct	ct	ct	ct	ct	ct	ct	ct	ct	ct	ct	ct	ct	ct	ct	ct	ct	ct	ct	ct
**Simulated sample***	4 °C	62.23	28.16	28.40	27.56	27.13	26.59	26.83	26.55	27.29	27.84	27.93	27.09	26.24	28.01	26.61	27.48	26.62	28.16	28.05	27.23	27.75	28.61	28.02	29.21	27.45
**Simulated sample***	4 °C	62.23	27.15	27.59	27.44	27.00	27.58	28.20	28.13	27.25	27.04	27.50	26.52	26.44	26.52	25.80	27.34	26.55	28.40	28.83	28.37	27.51	26.90	26.10	26.91	27.08
**Simulated sample***	4 °C	62.23	26.71	27.17	26.80	26.35	26.51	27.09	26.97	27.05	26.21	26.51	25.62	26.22	26.73	25.73	27.26	26.20	28.66	28.75	28.33	27.82	26.66	25.92	27.24	27.22
**Simulated sample***	24-27 °C	62.23	27.20	27.39	27.02	26.76	26.31	26.93	26.82	27.19	28.67	29.00	28.42	30.54	31.33	30.71	32.13	31.87	31.28	31.90	31.62	31.73	31.38	30.84	31.84	31.77
**Simulated sample***	24-27 °C	62.23	27.49	27.79	27.25	26.77	26.40	25.93	24.79	27.44	30.62	30.34	29.14	30.95	29.67	29.11	30.68	31.76	32.36	32.47	32.05	31.29	29.42	28.23	29.61	31.88
**Simulated sample***	24-27 °C	62.23	25.60	25.67	25.31	26.28	26.61	27.17	27.12	27.75	28.42	28.87	28.09	30.62	30.49	30.28	31.70	31.28	30.13	29.88	28.78	31.69	30.81	30.09	31.60	31.99
**Simulated sample***	37°C	62.23	26.13	26.37	26.24	25.45	26.89	27.63	28.02	30.49	33.66	34.03	33.44	33.74	32.48	31.27	33.02	34.28	37.10	35.89	36.28	36.94	31.15	30.23	31.96	32.02
**Simulated sample***	37°C	62.23	25.15	25.97	25.63	26.18	28.13	28.49	28.47	29.06	31.80	32.17	31.14	32.53	31.19	30.60	32.57	36.63	35.32	35.68	36.01	36.34	32.67	30.58	32.00	33.28
**Simulated sample***	37°C	62.23	26.11	26.24	25.93	26.47	27.36	27.29	26.89	29.14	29.37	29.53	28.79	33.51	34.10	32.36	33.10	34.13	33.28	33.31	32.95	35.95	31.98	30.08	31.04	33.80
**GARG medium***	4 °C	62.23	23.09	23.54	23.28	33.44	23.38	23.69	23.42	32.86	25.01	24.99	24.20	35.04	23.38	22.76	24.15	35.30	27.29	27.28	27.14	36.87	26.46	25.56	27.07	36.48
**GARG medium***	4 °C	62.23	23.28	23.65	23.35	34.14	23.02	23.13	22.70	32.88	25.25	25.22	24.54	34.88	24.99	24.17	25.47	35.69	28.61	28.71	28.29	37.91	28.11	27.27	28.89	38.21
**GARG medium***	4 °C	62.23	22.94	23.34	22.97	33.47	22.66	23.00	22.60	32.68	25.73	25.90	25.10	35.61	25.93	25.01	26.34	35.92	26.33	26.25	26.13	36.61	26.00	25.17	26.29	35.68
**Simulated sample**	24-27 °C		ND	ND	ND	26.58	ND	ND	ND	27.38	ND	ND	ND	29.00	ND	ND	ND	30.60	ND	ND	ND	31.21	ND	ND	ND	30.98
**GARG medium**	24-27 °C		ND	ND	ND	ND	ND	ND	ND	ND	ND	ND	ND	ND	ND	ND	ND	ND	ND	ND	ND	ND	ND	ND	ND	ND
**PC PCR**			32.18	30.05	29.66	27.83	33.41	30.06	30.10	27.77	33.64	32.04	30.42	28.20	33.58	30.17	30.83	27.55	34.54	32.78	31.36	29.26	34.04	29.90	30.92	28.24
**NTC PCR**			ND	ND	ND	ND	ND	ND	ND	ND	ND	ND	ND	ND	ND	ND	ND	ND	ND	ND	ND	ND	ND	ND	ND	ND

* Simulated sample/GARG medium spiked with SARS-CoV-2

E gene – *E* gene of SARS-CoV-2, *N* gene – *N* gene of SARS-CoV-2, *RdRP* - gene for RNA-dependent RNA polymerase of SARS-CoV-2, *GAPDH* – gene for human glyceraldehyde-3-phosphate dehydrogenase (internal control), ND - not detected, NTC PCR – negative template control of polymerase chain reaction, PC PCR - positive control of polymerase chain reaction, ct – cycle threshold value.

Article: Evaluation of non-invasive gargle lavage sampling for the detection of SARS-CoV-2 using rRT-PCR or antigen assay

Journal name: European Journal of Clinical Microbiology & Infectious Diseases

Author names: Hana Jaworek,[*,§,‡] Ondrej Bouska,[*, ‡] Vladimira Koudelakova,[*,§,#] Katerina Kubanova,[*] Petr Dzubak,[*,§, †] Rastislav Slavkovsky,[*] Branislav Siska,[*] Petr Pavlis,[*] Jana Vrbkova,[*] Marian Hajduch[*,§,†]

[*]Institute of Molecular and Translational Medicine, Faculty of Medicine and Dentistry, Palacky University, Olomouc, Czech Republic.

[§]Laboratory of Experimental Medicine, University Hospital Olomouc, Czech Republic

[†]Cancer Research Czech Republic, Olomouc, Czech Republic

[‡] *Both authors are equal.*

Corresponding author: Vladimira Koudelakova, MSc., Ph.D., ORCID: 0000-0001-5842-7478, Institute of Molecular and Translational Medicine, Faculty of Medicine and Dentistry, Palacky University, Hnevotinska 5, 775 15 Olomouc, Czech Republic.; vladimira.koudelakova@upol.cz
